# Direct Effects of the Janus Kinase Inhibitor Baricitinib on Sensory Neurons

**DOI:** 10.3390/ijms252211943

**Published:** 2024-11-06

**Authors:** Enrique Vazquez, Frank Richter, Gabriel Natura, Christian König, Annett Eitner, Hans-Georg Schaible

**Affiliations:** 1 Institute of Physiology 1/Neurophysiology, Jena University Hospital, Friedrich-Schiller-University, D-07740 Jena, Germany; enrique.vazquez-rodriguez@med.uni-jena.de (E.V.); frank.richter@med.uni-jena.de (F.R.); gabriel.natura@med.uni-jena.de (G.N.); christian.koenig@med.uni-jena.de (C.K.); 2 Department of Trauma, Hand and Reconstructive Surgery, Experimental Trauma Surgery, Jena University Hospital, Friedrich-Schiller-University, D-07740 Jena, Germany; annett.eitner@med.uni-jena.de

**Keywords:** baricitinib, Jak inhibitor, joint pain, nociceptor, neuronal Jak, DRG neurons, interleukin-6, pain sensitization, TNF, Stat3

## Abstract

Therapeutically, the Janus kinase (Jak) 1/Jak2 inhibitor baricitinib reduces the pathology of rheumatoid arthritis and may also reduce pain. Here, we investigated whether baricitinib directly affects joint nociceptors. We recorded action potentials from nociceptive C- and A∂-fibers of the normal and inflamed knee joint in anesthetized rats to monitor their responses to innocuous and noxious joint rotation. In isolated and cultured dorsal root ganglion (DRG) neurons, we examined Stat3 activation using Western blots and monitored excitability using patch-clamp recordings. Intra-articular injection of baricitinib did not alter C- and A∂-fiber responses to innocuous and noxious rotations of the normal knee but reduced C-fiber responses to these stimuli in inflamed joints. Baricitinib prevented the increase in C-fiber responses to joint rotation evoked by interleukin (IL)-6 plus soluble interleukin-6 receptor (sIL-6R) but not the increase evoked by TNF. In DRG neurons, baricitinib blocked Stat3 activation by hyper-IL-6, and baricitinib or the Stat3 inhibitor Sta21 prevented induction of hyperexcitability by IL-6 plus sIL-6R. Thus, neuronal Jaks are involved in the generation of C-fiber hyperexcitability induced by inflammation and IL-6. Pain reduction by baricitinib may result, at least in part, from direct effects on joint nociceptors.

## 1. Introduction

The pro-inflammatory cytokines tumor necrosis factor (TNF), interleukin (IL)-6, IL-1β, IL-17, and others generate and maintain inflammation in rheumatoid arthritis and related diseases [1]. They also contribute to pain, not only by promoting inflammation but also by directly activating cytokine receptors in sensory neurons and the central nervous system [2,3,4,5]. They induce neuronal hyperexcitability, called sensitization [3]. A sensitized nociceptive system is a hallmark of joint diseases such as rheumatoid arthritis and osteoarthritis [6]. Hyperexcitable nociceptive neurons fire more action potentials when stimulated, and their excitation threshold is lowered so that normally innocuous and non-painful stimuli, such as joint movements, are sufficient to evoke discharges and produce pain [7].

Some cytokines, such as IL-6 and IL-17, share a common cellular mechanism of transduction, the activation of intracellular Janus kinase (Jak) pathways. Upon cytokine binding to its membrane receptor, associated Jaks are phosphorylated, form dimers, and in turn phosphorylate the receptor and other protein substrates, therefore opening a series of possible signaling. By recruiting signal transducer and activator of transcription (Stat) proteins (transcription factors that enter the nucleus) to the activated receptor and phosphorylating them, Jaks directly gates a route to transcription [8]. Coupling of Jaks to other signaling pathways, such as phosphatidylinositol 3-kinase (PI3K) extracellular signal-*regulated* kinase (ERK), can mediate rapid cytokine effects [9].

The Jak1 and Jak2 inhibitor baricitinib competes with the ATP binding site of Jak and transiently and reversibly prevents Jak phosphorylation and activation, therefore disrupting downstream signaling [8]. Inhibition of Jak1 and Jak2 suppresses inflammation and bone resorption in several models of arthritis [10,11]. In clinical trials in patients with rheumatoid arthritis, baricitinib reduced inflammation as much as or more than neutralizing TNF. In addition, patients reported even greater pain relief with baricitinib than with a TNF blocker [12,13,14]. Since pro-inflammatory cytokines contribute to pain generation (see above), Jak inhibitors may contribute to pain reduction by inhibiting direct neuronal cytokine effects on nociceptive neurons.

In the present experiments, we tested the hypothesis that the neuronal activity of nociceptive sensory neurons can be influenced by baricitinib. First, we investigated in vivo whether the application of baricitinib to the knee joint affects the responses of joint nociceptors to innocuous and noxious knee movements under normal conditions and during acute inflammation. Second, we investigated whether intra-articular baricitinib affects the effect of IL-6 and TNF on joint nociceptors. Since IL-6 plus its soluble receptor (sIL-6R) causes long-lasting sensitization of joint nociceptors to mechanical stimuli [15], and since both Jak1 and Jak2 mediate cellular IL-6 effects [8], we investigated whether the sensitization of joint nociceptors by IL-6 is affected by baricitinib. For comparison, we tested whether the effect of TNF, which also sensitizes joint nociceptors to mechanical stimuli [16] but does not signal through the Jak/Stat pathway [8], is also affected by baricitinib. In addition, we tested whether baricitinib also alters neuronal excitability and/or the sensitizing effect of IL-6 + sIL-6R in isolated and cultured dorsal root ganglion (DRG) neurons.

## 2. Results

### 2.1. Effects of Baricitinib on Joint Nociceptor Responses to Mechanical Joint Stimulation In Vivo

**Figure 1:**
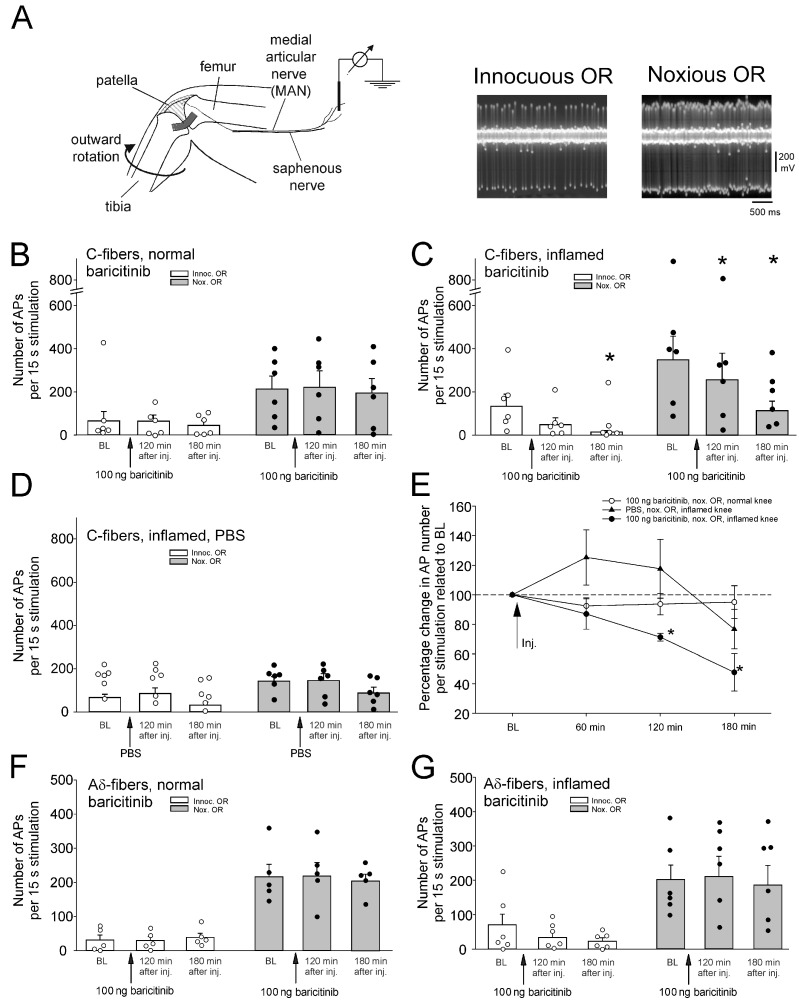
Effect of intra-articular injection of baricitinib on joint nociceptor responses to innocuous and noxious outward rotation (OR) of the normal and inflamed knee joint. (**A**) Experimental setup for in vivo recordings from joint nociceptors in anesthetized rats (**left**) and typical action potentials of a single nerve fiber evoked by innocuous and noxious OR (**right**). (**B**) No change in response magnitude of normal knee joint C-fibers (*n* = 6) to innocuous and noxious OR by baricitinib application (arrows). Fibers were continuously recorded for 180 min. (**C**) Reduction of C-fiber responses (*n* = 6) of the acutely inflamed joint to innocuous and noxious OR by baricitinib application. (**D**) No significant change of C-fiber responses (*n* = 6) to innocuous and noxious OR of the acutely inflamed joint by PBS. (**E**) Summary of the effects shown in (**B**–**D**). Baseline values before baricitinib or PBS injection were normalized to 100%. (**F**) No change in A∂-fiber responses (*n* = 5) of the normal knee joint to innocuous and noxious OR by baricitinib. (**G**) No change in A∂-fiber responses (*n* = 6) of the inflamed knee joint to innocuous and noxious OR by baricitinib. White circles and black bullets indicate single data points from individual fibers. * *p* < 0.05, Wilcoxon matched-pairs signed-rank test.

We first investigated whether injection of baricitinib into the normal knee joint altered nociceptor responses to outward rotation (OR). Figure 1A shows the experimental setup. Single joint nociceptors were isolated from small nerve bundles of the saphenous nerve, and action potentials were recorded extracellularly. Nerve fibers supplying the knee joint were identified by localizing a receptive field in the joint by probing the exposed joint with the tip of a glass rod and by electrical stimulation of the receptive field. Responses to innocuous and noxious OR of the joint were monitored. Typically, nociceptors show either no or a small response to innocuous OR and a marked response to noxious OR (see specimens in Figure 1A). To test the effect of compounds, single fibers (one fiber per experiment) were recorded continuously before and after intra-articular injection of compounds.

Injection of baricitinib (100 ng in 100 µL, corresponding to an initial concentration of 2.7 µM) into the normal knee joint did not alter the magnitude of C-fiber responses to innocuous and noxious OR over 180 min (Figure 1B). In further experiments, we started recordings from nerve fibers at 7–11 h after induction of knee inflammation, which sensitizes joint nociceptors within hours [7]. Intra-articular injection of baricitinib (100 ng in 100 µL) into the inflamed knee significantly reduced the magnitude of C-fiber responses within 3 h (Figure 1C). Application of PBS into the inflamed joint did not significantly alter C-fiber responses (Figure 1D). Figure 1E compares the data for noxious ORs, shown in Figure 1B–D. Baseline responses before knee injection were normalized to 100%. Only injection of baricitinib into the inflamed joint caused a significant reduction in the responses to innocuous and noxious OR.

Joint A∂ fiber responses of the joint were not altered by baricitinib in either the normal joint (Figure 1F) or the inflamed joint (Figure 1G).

### 2.2. Baricitinib Suppresses Joint Nociceptor Sensitization by IL-6 + sIL-6R but Not by TNF

The potent stimulation of neurons by IL-6 is mediated by IL-6-trans-signaling [15,17]. IL-6 forms complexes with the soluble IL-6 receptor (sIL-6R) that activate the signal transduction unit glycoprotein 130 (gp130), which is ubiquitously expressed in the cell membrane and present in more than 90% of DRG neurons [18]. Intra-articular injection of IL-6 + sIL-6R into the knee joint increases the responses of knee joint C-fibers [15] and spinal cord neurons with knee input to mechanical stimulation of the joint [19]. Since IL-6 activates Jak1, Jak2, and Stat3 [8], we tested whether baricitinib (100 ng in 100 µL) affects IL-6 signaling in joint nociceptors.

**Figure 2:**
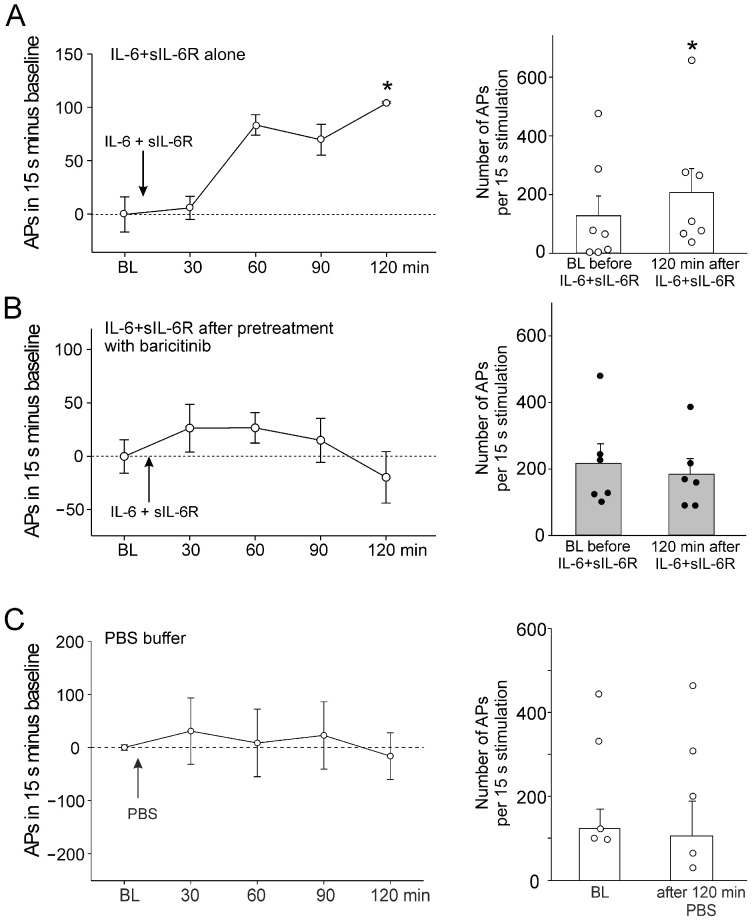
Effect of baricitinib on induction of C-fiber hyperexcitability by IL-6 + sIL-6R. (**A**) Persistent increase in C-fiber responses (*n* = 7) to noxious OR by intra-articular injection of IL-6 + sIL-6R (arrow). The right columns show responses before and 120 min after IL-6 + sIL-6R. (**B**) No significant increase in responses of C-fibers (*n* = 6) to noxious OR by IL-6 + sIL-6R after pretreatment with baricitinib for 60 min. (**C**) No change in C-fiber responses (*n* = 5) to noxious OR by intra-articular injection of PBS. BL, baseline (set to zero in graphs of the left), White circles and black bullets indicate single data points from individual fibers. * *p* < 0.05, Wilcoxon matched-pairs signed-rank test.

In C-fibers, a single intra-articular injection of IL-6 + sIL-6R significantly increased the responses to noxious OR. Figure 2A, left, shows the increase in responses compared to the normalized baseline (set to zero). Figure 2A, right, shows the magnitude of the responses before and 120 min after IL-6 + sIL-6R injection. After intra-articular injection of baricitinib, the subsequent intra-articular injection of IL-6 + sIL-6R did not significantly affect the responses to noxious OR (Figure 2B).

Intra-articular injection of PBS did not alter the responsiveness (Figure 2C). Thus, the sensitization of neurons by IL-6 + sIL-6R was blocked by baricitinib. Because IL-6 + sIL-6R does not significantly affect the responses of A∂ fibers to mechanical stimulation [15], these fibers were not tested in the same way as C-fibers.

**Figure 3:**
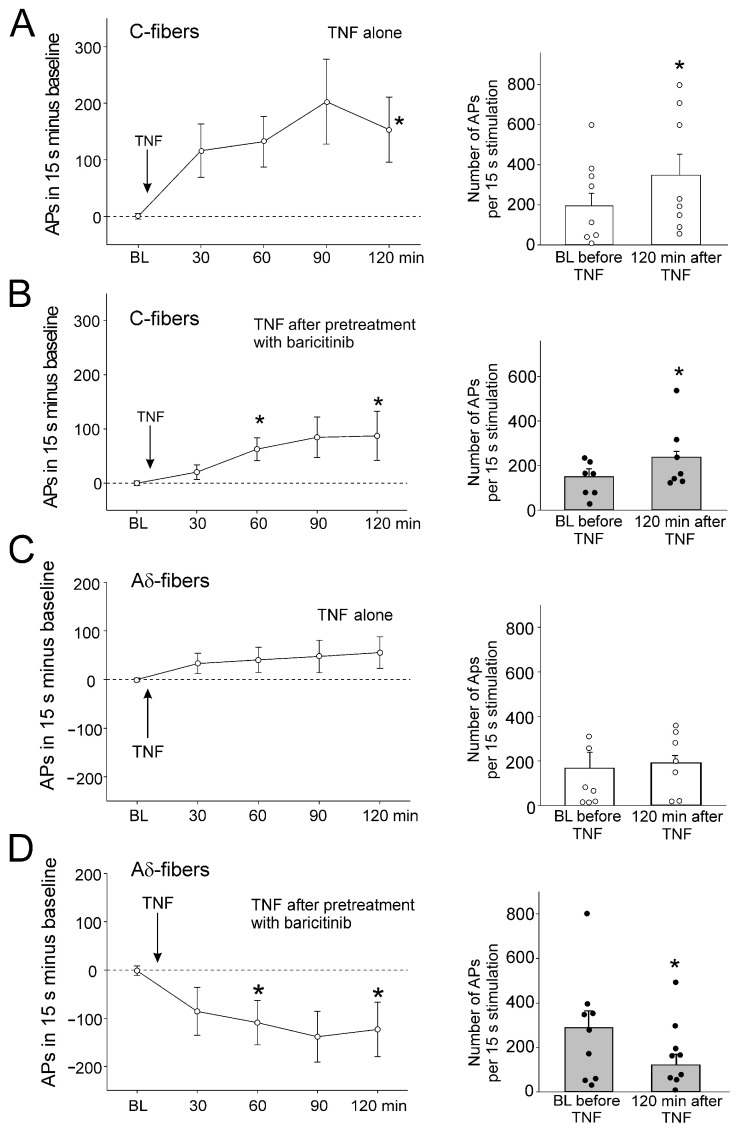
Effect of baricitinib on TNF-induced hyperexcitability of C-fibers. (**A**) Persistent increase in C-fiber responses (*n* = 8) to noxious OR by intra-articular injection of TNF (arrow). The right columns show responses before and 120 min after TNF. (**B**) Significant increase in responses of C-fibers (*n* = 7) to noxious OR by TNF after pretreatment of fibers with baricitinib for 60 min. (**C**) No significant change in responses of A∂-fibers (*n* = 7) to noxious OR by intra-articular injection of TNF. (**D**) Significant reduction of A∂-fiber responses (*n* = 8) to TNF after pretreatment with baricitinib for 60 min. White circles and black bullets indicate single data points from individual fibers. BL, baseline, * *p* < 0.05, Wilcoxon matched-pairs signed-rank test.

Intra-articular injection of TNF significantly increased C-fiber responses to noxious rotation (Figure 3A). Injection of baricitinib did not prevent the TNF-induced increase in C-fiber responses, as the increase was still significant (Figure 3B). TNF did not significantly increase A∂ fiber responses to noxious OR (Figure 3C). Interestingly, A∂ fiber responses were significantly reduced after intra-articular injection of baricitinib (Figure 3D).

### 2.3. Effects of Bariticinib on Stat3 Activation in Isolated DRG Neurons

**Figure 4:**
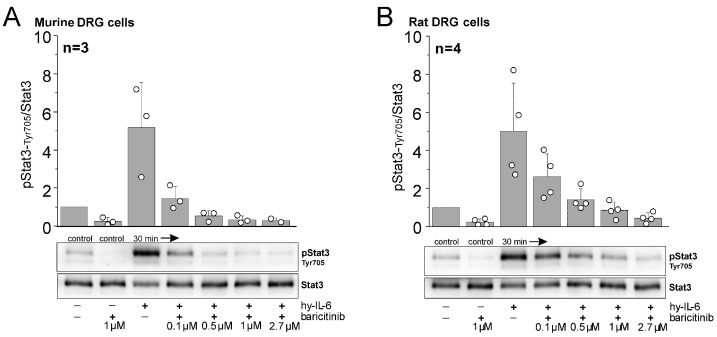
Inhibitory effect of baricitinib on IL-6-dependent phosphorylation of pStat3-Tyr705. Representative Western blot analyses of mouse (**A**) and rat DRG cells (**B**) stimulated with hyper-IL-6 (hy-IL-6), a recombinant fusion protein of IL-6 and sIL-6R, for 30 min in the absence or presence of indicated concentrations of baricitinib. Bar graphs represent densitometric ratios of activated Stat3 at tyrosine residue 705 (pStat3-Tyr705) to total Stat3 protein normalized to vehicle-treated controls for mouse (from *n* = 3 mice) and rat DRG cells (from *n* = 4 rats). White circles indicate data points from individual animals.

Western blot experiments on isolated, cultured DRG neurons from mice and rats showed some basal phosphorylation of Stat3 (pStat3), but stimulation with hyper-IL-6, a fusion molecule of IL-6 and sIL-6R [20], strongly activated Stat3 (Figure 4A,B). Here, the ratio of pStat3/Stat3 is shown 30 min after stimulation, but strong Stat3 activation is already seen 15 min after stimulation, and initial increases begin 5 min after stimulation [21]. Baricitinib inhibited both basal Stat3 activation and hyper-IL-6-induced Stat3 activation in a dose-dependent manner. These data clearly demonstrate that DRG cells are a target for baricitinib.

Baricitinib at 2.7 µM (corresponding to the starting dose of baricitinib in the rat in vivo experiments) effectively blocked Stat3 activation in DRG cells from rats. In mouse cells, Stat3 activation was blocked by baricitinib at 1 µM. Therefore, this concentration was used in the recordings from isolated mouse DRG neurons (see Section 2.4).

### 2.4. Effect of Baricitinib on the Induction of Hyperexcitability in Isolated DRG Neurons by IL-6 + sIL-6R

**Figure 5:**
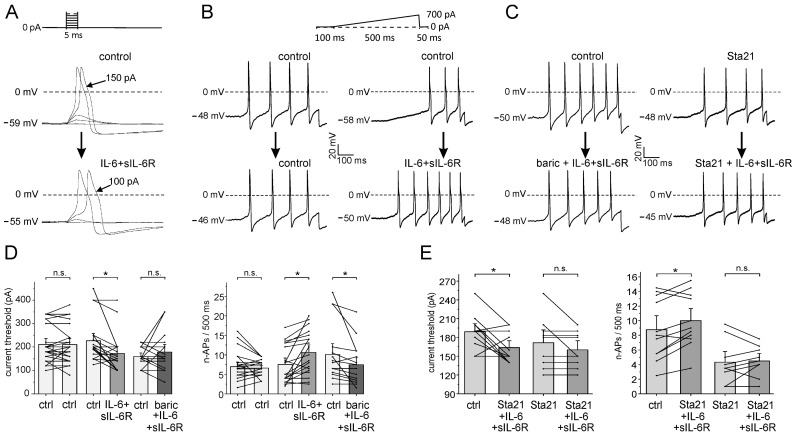
Effect of baricitinib and Sta21 on IL-6 + sIL-6R-evoked sensitization of isolated DRG neurons. (**A**) Determination of current threshold for AP elicitation (decrease from 150 pA to 100 pA by IL-6 + sIL-6R). (**B**,**C**) Specimens of single neurons tested for the number of APs (n-APs) elicited during ramp current injection before and after the application of different compounds. (**D**) (**Left**): current threshold in control neurons (ctrl-ctrl, *n* = 22), the effect of IL-6+sIL6R (ctrl-IL-6 + sIL-6R, *n* = 19), the effect of co-application of IL-6 + sIL-6R and 1.0 µM baricitinib (ctrl-baric+ IL-6 + sIL-6R, *n* = 17). (**Right**): n-APs in control neurons (*n* = 22), effect of IL-6 + sIL-6R on n-APs (*n* = 19), effect of IL-6 + sIL-6R plus 1.0 µM baricitinib co-application on n-APs (*n* = 17). (**E**) (**Left**): Effect of IL-6 + sIL-6R and Sta21 co-application (*n* = 11) and IL-6 + sIL-6R after Sta21 preincubation (*n* = 9) on current threshold. (**Right**): Effect of IL-6 + sIL-6R and Sta21 co-application (*n* = 11) and IL-6 + sIL-6R after Sta21 preincubation (*n* = 9) on n-APs. n.s., non significant, * *p* < 0.05, paired *t*-test.

Using whole-cell patch-clamp recordings from small- to medium-sized neurons (most of which are nociceptive), we investigated whether baricitinib interferes with the effect of IL-6 + sIL-6R on the neuronal excitability of isolated and cultured DRG neurons. Using a step protocol, we tested at what current intensity action potentials (APs) are elicited (Figure 5A). Using a ramp protocol, we tested how many APs (n-APs, number of APs) are elicited during the current ramp. Figure 5B,C show typical specimens. Repeated stimulation without bath application of a compound did not change n-APs (Figure 5B, column 1, control—control). Application of IL-6 + sIL-6R after superfusion with buffer solution (Figure 5B, column 2, control—IL-6 + sIL-6R) increased the induced n-APs within 3 min. When IL-6 + sIL-6R was applied together with baricitinib, n-APs were not increased (Figure 5C, column 1). After preincubation of the Stat3 inhibitor Sta21 for at least 30 min, only one additional AP was added by co-application of IL-6 + sIL-6R and Sta21 (Figure 5C, column 2).

Control neurons were repeatedly tested without compound application. On average, neither current threshold (Figure 5D, columns 1 and 2) nor n-APs (Figure 5D, columns 7 and 8) were significantly altered. Application of IL-6 + sIL-6R significantly decreased the current threshold (Figure 5D, columns 3 and 4) and significantly increased n-APs (Figure 5D, columns 9 and 10) within 5–8 min. When IL-6 + sIL-6R was co-administered with baricitinib 1.0 µM, the current threshold was not significantly changed (Figure 5D, columns 5 and 6), and n-APs was significantly reduced (Figure 5D, columns 9 and 10). The application of baricitinib (1.0 µM) alone did not affect the current threshold and n-APs (Supplementary Figure S1).

Since Stat3 is downstream of Jak activation, we also examined whether the Stat3 inhibitor Sta21 affected the response of neurons to IL-6 + sIL-6R. Simultaneous application of IL-6 + sIL-6R with Sta21 did not prevent the IL-6 + sIL-6R-induced decrease in the current threshold (Figure 5E, columns 1 and 2) and the increase in elicited n-APs (Figure 5E, columns 5 and 6). However, after preincubation with Sta21 for at least 30 min, IL-6 + sIL-6R did not decrease the current threshold (Figure 5E, columns 3 and 4) and did not increase n-APs (Figure 5E, columns 7 and 8).

**Figure 6:**
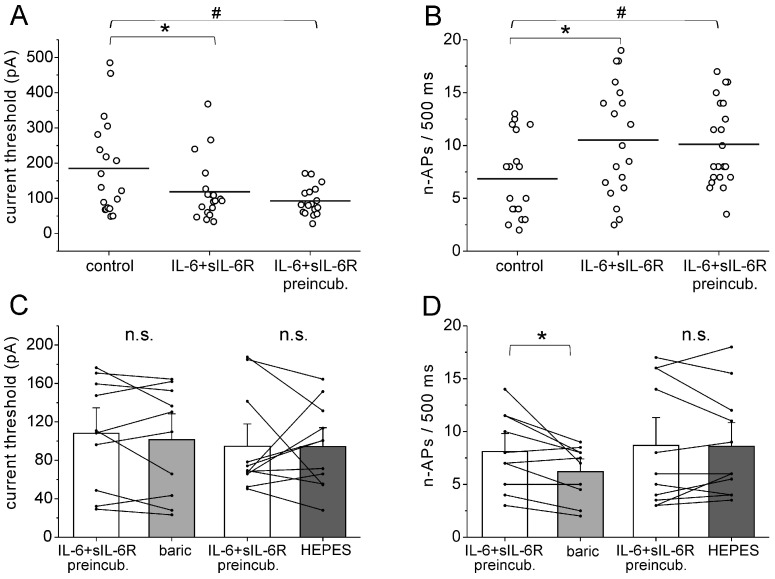
Effect of baricitinib on established hyperexcitability of isolated DRG neurons. (**A**) Current threshold for AP elicitation in untreated DRG neurons (*n* = 19), in neurons after short-term application of IL-6 + sIL-6R (*n* = 19, same neurons as control neurons, paired analysis), and in neurons after long-term preincubation with IL-6 + sIL-6R (*n* = 21). Current threshold was measured by ramp current injection. (**B**) Number of evoked APs (n-APs) during the current ramp in untreated DRG neurons (*n* = 19), in neurons after short-term application of IL-6 + sIL-6R (*n* = 19, same neurons as control neurons, paired analysis), and in neurons after long-term preincubation with IL-6 + sIL-6R (*n* = 21). (**C**) No significant change in current threshold by application of baricitinib (*n* = 10) or HEPES solution (*n* = 11). (**D**) Reduction of n-APs in neurons after application of baricitinib (*n* = 10), but not HEPES solution (*n* = 11). n.s., non significant, * *p* < 0.05, paired *t*-test, ^#^ unpaired *t*-test.

In addition, we investigated whether baricitinib reduces IL-6 + sIL-6R-induced neuronal hyperexcitability once it is established. In these experiments, cells were preincubated with IL-6 + sIL-6R for 30 min before the current threshold, and the elicited n-APs were measured. The IL-6 + sIL-6R solution was then replaced by a solution containing baricitinib or HEPES solution. Evaluation of the current threshold for AP elicitation with the current ramp showed that the reduction of the current threshold was significant after both short-term application and preincubation of IL-6 + sIL-6R (Figure 6A), and the increase of n-APs was also stable (Figure 6B). After preincubation with IL-6 + sIL-6R for 30 min, the current threshold was not changed by the application of baricitinib or HEPES solution (Figure 6C). However, n-APs were significantly reduced by baricitinib but not by the HEPES solution (Figure 6D). Thus, baricitinib reduced a parameter of established neuronal hyperexcitability.

## 3. Discussion

This study demonstrates a direct effect of the Jak1/Jak2 inhibitor baricitinib on sensory neurons. In vivo, baricitinib did not affect the responses of joint nociceptors to noxious stimuli of the normal knee joint but reduced the responses of joint nociceptors to mechanical stimulation of the inflamed knee joint. Baricitinib abolished the sensitizing effect of IL-6 + sIL-6R on C-fiber joint nociceptors but not the sensitizing effect of TNF, which is not linked to Jak/Stat pathways. In isolated DRG neurons, baricitinib alone did not significantly alter neuronal excitability but prevented the induction of neuronal hyperexcitability by IL-6 + sIL-6R. In conclusion, the data show that baricitinib has potent effects on sensory neurons, suggesting that inhibition of Jak pathways in sensory neurons significantly contributes to pain attenuation after baricitinib application.

Most joint nociceptors are mechanosensitive. High-intensity (painful) mechanical stimuli open mechanosensitive ion channels in the cell membrane, such as TRPV4 ion channels in nociceptive C-fibers [22] and Piezo 2 ion channels [23] and cause a local depolarization of the sensory ending by inducing a cation influx from the extracellular space into the cytoplasm. A sufficiently strong local depolarization triggers the formation of action potentials by opening voltage-gated Na^+^ channels. Since baricitinib did not affect responses to noxious stimuli in the absence of inflammation, responses to noxious mechanical stimuli per se do not appear to involve Jak activation.

Importantly, however, nociceptor mechanosensitivity increases during inflammation. The mechanical threshold for neuronal activation is lowered, and noxious mechanical stimuli evoke stronger responses. Nociceptor sensitization contributes to the mechanical hyperalgesia (pain during movements and palpation) typical of joint disease [6,7,24,25]. In C-fibers, sensitization to mechanical stimuli is produced by inflammatory mediators. They act on membrane receptors of sensory neurons and induce intracellular signaling cascades that subsequently increase the sensitivity of mechanosensitive ion channels, e.g., by phosphorylation [26]. Such signaling cascades also increase neuronal excitability by affecting voltage-gated Na^+^ channels [26,27,28]. The reduction of C-fiber responses in the inflamed joint by baricitinib suggests that Jak pathways are involved in enhanced nociceptor responsiveness during inflammation.

Baricitinib completely blocked IL-6 + sIL-6R-induced C-fiber sensitization in vivo. Although IL-6 can also activate non-neuronal cells such as chondrocytes [29] and synoviocytes [30,31], neurons themselves are also a target of baricitinib. Baricitinib blocked Stat3 activation in isolated DRG cells, and patch-clamp recordings showed that baricitinib and Sta21 antagonized the induction of hyperexcitability by IL-6 + sIL-6R in isolated DRG neurons. Baricitinib also partially reduced established IL-6 + sIL-6R-induced hyperexcitability in isolated DRG neurons.

A comprehensive analysis of mRNA in DRGs showed that baricitinib restored gene expression alterations in DRGs induced by collagen antibody-induced arthritis, CAIA [11].

Neutralization of either IL-6 signaling by sgp130 [32] or IL-17A signaling by anti-IL-17 antibody [33] reduced pain-related behaviors in arthritis models, and IL-17A knockout mice showed significantly less mechanical hyperalgesia than wild-type mice in antigen-induced arthritis despite similar levels of inflammation [34]. Sensitization of joint nociceptors by IL-17A was not blocked by etanercept (neutralizing TNF) or sgp130 [33]. However, intracellular Jak/Stat pathways are shared by different cytokines. For example, IL-6 signaling activates Jak1, Jak2, TYK2 and Stat3, and IL-17A activates Jak2, Stat1 and Stat3 [8]. Therefore, Jak inhibitors may more effectively inhibit the intracellular signaling that leads to neuronal sensitization than neutralization of individual cytokines (or cytokine receptors) by antibodies. However, Jak/Stat pathways may also mediate the effects of anti-inflammatory cytokines such as IL-4 and IL-10 [8], and some cytokines, such as TNF and IL-1β, are not linked to Jak/Stat pathways [8]. Here, we show that the sensitizing effect of TNF on C-fibers was not abolished by baricitinib. The reduction of responses after the co-administration of TNF and baricitinib in A∂ fibers was surprising but cannot be explained currently. Finally, neuronal sensitization is also mediated by a large family of G protein-coupled receptors, for example by prostaglandins [35]. The reduction of C-fiber responses to mechanical stimulation of the inflamed joint by baricitinib suggests that sustained activity of Jak/Stat pathways significantly contributes to mechanical sensitization.

The precise cellular mechanisms downstream of Jak activation remain to be elucidated. It is unclear whether all downstream effects of Jaks are mediated by Stat activation. Stats have been mainly characterized as transcription factors that are transported into the nucleus and modify gene transcription. Since bath application of IL-6 + sIL-6R increases neuronal excitability in isolated neurons within 3–5 min, it is questionable whether such a rapid effect involves the nucleus. Simultaneous co-incubation with the Stat3 inhibitor Sta21 did not prevent IL-6 + sIL-6R-induced hyperexcitability. However, preincubation of Sta21 (here for about 30 min) prevented the increase in neuronal excitability by IL-6 + sIL-6R. We, therefore, suggest that the rapid initial effects of cytokines are due to the coupling of Jaks to signaling pathways such as PI3K and ERK [9]. However, the Sta21 effect (upon preincubation) suggests that Stat3 activation finally plays a role in the IL-6 + sIL-6R-induced hyperexcitability. Notably, the exact targets of neuronal signaling need to be further investigated. Presumably, various ion channels in the cell membrane, including ion channels of mechanotransduction (see above) and voltage-gated ion channels, are ultimate targets of intracellular signaling cascades of cytokines and specific Jaks and Stats. Even mitochondria have been identified as a target of Stat3 [36], suggesting that cytokines regulate neuronal energy metabolism, which may influence excitability.

Taylor et al. [12] documented a significant reduction in pain in patients with rheumatoid arthritis by baricitinib after the first week, with further pain reduction in the following weeks (see also [14]). Presumably, the time of the first pain reduction was not systematically analyzed. For example, neutralization of TNF can begin to reduce pain in experimental arthritis and in patients within one day [37,38]. Pain in patients with rheumatoid arthritis has a complex pathogenesis, including sensitization of peripheral nociceptors, central sensitization (spinal cord and brain), and the development of a neuropathic component in some patients [6,8,11,39]. The (partial) reversal of these changes may take different times, thus explaining the progression of pain relief over time. Since cytokines such as IL-6 are involved in both inflammatory and neuropathic pain [32,40,41], Jak inhibition may reduce pain at different time points.

The present study focused on the direct inhibitory effects of baricitinib on sensory neurons. Pain reduction by baricitinib may also involve mechanisms in the central nervous system. Nociceptors, sensitized by inflammation, induce a state of spinal sensitization resulting from increased synaptic processing of peripheral input and activation of microglial and astroglial cells [4,42]. In CAIA, baricitinib reduced behavioral allodynia and prevented spinal microglial and astroglial activation, possibly by reducing colony-stimulating factor 1 (CSF-1) expressed and released spinally by DRG neurons [11]. Oral baricitinib also downregulated microglial and astroglial activation in the spinal cord during experimental autoimmune encephalomyelitis EAE, a model of multiple sclerosis [43]. It is unclear whether baricitinib can cross the blood-brain barrier (for discussion, see [44]), but in collagen-induced arthritis (CIA), oral baricitinib reduced microglial and Stat3 activation in the area postrema, where the blood-brain barrier is weak [45]. Jak inhibitors reduced bursting activity in cultured cortical neurons [46], suggesting that cortical neurons may also be affected by Jak inhibition.

In conclusion, the present study demonstrated a direct effect of baricitinib on sensory neurons. Therefore, it is likely that the reduction of pain after baricitinib treatment is not only due to the amelioration of pathological processes but also to the inhibition of direct cytokine actions on sensory neurons and the subsequent reduction of pain processing in the central nervous system. Neuronal effects of baricitinib may also be involved in the reduction of pathological changes as the nervous system promotes inflammatory processes through the release of neuropeptides from sensory neurons (neurogenic inflammation) and the sympathetic nervous system. For example, IL-6 signaling in sensory neurons contributes to the development of swelling in antigen-induced arthritis because it stimulates the release of CGRP, a vasodilatory peptide, from sensory neurons [47].

Several limitations of the present study should be noted. Our experimental approach clearly demonstrated that sensory neurons are a target of baricitinib and that baricitinib has analgesic potential. However, we did not test the analgesic potential of baricitinib in long-term inflammation, nor did we test the analgesic potential of baricitinib in neuropathic pain states. Further experimental and clinical studies are needed to investigate the analgesic potential of baricitinib in the clinical setting. While baricitinib is currently used in the treatment of rheumatoid arthritis, the demonstration that sensory neurons are a target of baricitinib may stimulate interest in investigating the analgesic potential of baricitinib in other painful conditions. However, similar to biologics that neutralize cytokines, Jak inhibitors may also have safety issues, such as infections resulting from suppression of the immune system [14].

## 4. Materials and Methods

### 4.1. Nerve Fiber Recordings

Adult male Wistar rats (53 with normal knees, 18 with acute knee inflammation, age >90 days, body weight 300–400 g) were anesthetized with 100 mg/kg sodium thiopentone (Altana, Konstanz, Germany) i.p., areflexia was maintained with additional doses (20 mg/kg). Mean arterial blood pressure, electrocardiogram, and body temperature were monitored, and rats breathed spontaneously. The right thigh was exposed from the knee joint to the groin, a clamp secured the right femur, and the right hind paw was secured in a shoe-like holder that allowed calibrated rotation of the lower leg at the knee joint. An innocuous outward torque (Innoc. OR, 20 mNm, 15 s) and a noxious outward torque (Nox. OR, 40 mNm, 15 s) were applied manually and adjusted using the display of a torque meter (MVD2510, HBM Hottinger-Baldwin, Darmstadt, Germany).

Action potentials (APs) were recorded from single fibers of the medial articular nerve with receptive fields in the knee joint using platinum wire electrodes. The local mechanical threshold in the receptive field was determined using a glass rod and calibrated von Frey hairs (1.6–52 g). To determine the nerve fiber conduction velocity (C: ≤1.25 m/s, A∂: 1.25–10 m/s, Aβ: ≥10 m/s), the receptive field was stimulated with bipolar electrodes (0.5 ms pulses of 1–10 V). The AP signals were transferred to a PC (DAB 1200 interface card, Microstar Laboratories Inc., Bellevue, WA, USA) to construct peristimulus time histograms using the spike/spidi software (SpiKE program, version 2.91, for AP recording, SPIDI program, version 2.90, for evaluation; developed by C. Forster, University of Erlangen-Nurnberg, Erlangen, Germany) [48].

Recordings were made in blocks of 15 min: 3 × innocuous, 3 × noxious OR at 1 min intervals. The number of APs evoked quantified the responses. After monitoring baseline responses (typically the first 4 blocks of testing), 100 µL of the test substance was injected intra-articularly, and testing continued for 3 h. Some rats were recorded 7–11 h after induction of inflammation (see below). Finally, the rats were killed by intravenous injection of 1.0 mL sodium thiopentone.

For knee injection (volume 100 µL), we used baricitinib (100 ng, #7222, Tocris, Wiesbaden-Norderstedt, Germany), IL-6 (20 ng, #CYT-213, Prospec, Ness Ziona, Israel), sIL-6R (20 ng, #CYT-286, Prospec), all dissolved in PBS, and recombinant rat TNF (5 ng, #CYT-393, Prospec), dissolved in NaCl solution. Data from animals receiving the same treatment were averaged. Values are expressed as mean ± SEM.

### 4.2. Induction of Acute Inflammation by Intra-Articular Kaolin/Carrageenan Injection

Acute inflammation was induced in the right knee joint by injection of 70 μL of a kaolin suspension (Sigma, Taufkirchen, Germany; 4%, in H_2_O) followed 15 min later by 70 μL of a carrageenan solution (Sigma; 2%, in H_2_O) into the joint cavity [15]. Recordings were made 7–11 h after induction of inflammation.

### 4.3. Primary Culture of DRG Neurons

Adult C57BL/6J mice and Wistar rats were killed by CO_2_. Dorsal root ganglia (DRGs) were dissected from all spinal cord segments and collected in Ham’s F12 medium (BioWest, Nuaillé, France). Digestion followed by collagenase type II in Ham’s F12 (240 U/mL, Sigma, Munich, Germany) for 60 min (mice) or 90 min (rats) and, after washing with Ca^2+^- and Mg^2+^-free PBS (Gibco, Loughborough, UK), trypsin (10,000 U/mL, Sigma) for 10 min (mice) or 15 min (rats) at 37 °C. DRG cells were suspended in Ham’s F12, dispersed by mechanical trituration with a fire-polished Pasteur pipette, and collected by centrifugation (500× *g* for 8 min). Cell pellets were suspended in Ham’s F12 and 10% heat-inactivated horse serum (Sigma) supplemented with 1 mM glutamine (Sigma), 1% PenStrep (Thermo Scientific, Waltham, MA, USA), and 50 ng/mL nerve growth factor (Enzo, Lörrach, Germany). They were then seeded onto poly-L-Lysine (50 µg/mL, Sigma)- coated surfaces of either coverslips for patch-clamp experiments or 12-well cell culture plates (Thermo Scientific) for stimulation experiments. Cells were placed at 37 °C in a humidified incubator gassed with 5% CO_2_ and air, and cells were used within 30 h of culture.

### 4.4. Intracellular Signaling, SDS-PAGE, Western Blot

Stimulation experiments were performed on independent DRG cell preparations and repeated at least three times. For signaling analysis, mouse and rat DRG cells were washed with pure DMEM (BioWest, Nuaillé, France). Cells were treated with 25 ng/mL hyper-IL-6 (hy-IL-6, a recombinant fusion protein of human IL-6 and its soluble IL-6 receptor, #8954-SR, R&D Systems, Minneapolis, MN, USA) dissolved in DMEM for 30 min. When hy-IL-6 was combined with different concentrations of baricitinib (100 nM, 500 nM, 1 µM, 2.7 µM, #cay16707-25, Cayman Chemicals, Ann Arbor, MI, USA), baricitinib was preincubated for 20 min and always present throughout the hy-IL-6 treatment. The reaction was stopped on ice, the medium was aspirated, and the cells were scraped and harvested with RIPA lysis buffer [20 mM Tris-HCl pH 7.4, 150 mM NaCl, 1 mM EDTA, 1 mM EGTA, 1 mM β-glycerophosphate, 2.5 mM sodium pyrophosphate, 1% sodium deoxycholate, 1% NP-40 freshly supplemented with protease inhibitor cocktail tablets (Roche, Mannheim, Germany)]. After a freeze-thaw cycle, cell lysates were centrifuged, and Laemmli loading buffer was added to supernatants. For protein analysis, protein samples were separated on 10% PAGE gels (Serva, Heidelberg, Germany) at 125 V and transferred to a PVDF membrane (Millipore, Billerica, MA, USA). Immunoblotting was performed using anti-phospho-Stat3-Tyr705 (#9145, RRID:AB_2491009, Cell Signaling Technology, Danvers, MA, USA) and anti-Stat3 (#4904, RRID:AB_331269, Cell Signaling Technology). Signals were visualized with HRP-conjugated secondary antibody (#5220-0336, RRID:AB_2721169, SeraCare, Milford, MA, USA) and an enhanced chemiluminescence reaction (Thermo Fisher) using a CCD camera system and GeneSnap 7.12 gel documentation software (RRID:SCR_014249, Synoptics, Cambridge, UK). Densitometry of the corresponding band intensities was performed using Image J 1.52a software (RRID:SCR_003070).

### 4.5. Patch-Clamp Recordings from Isolated and Cultured DRG Neurons

Recordings were made from small- and medium-sized DRG neurons (capacitances between 18 and 30 pF, corresponding to cells ≤ 30 μm diameter) from mice at room temperature using an EPC-10 USB-double amplifier and HEKA PATCHMASTER 2.65 software (HEKA Electronics, Lambrecht, Germany). Cells were used 12–24 h after plating.

The bath was perfused with HEPES solution [control; (in mM): 118 NaCl, 5 KCl, 2 CaCl_2_, 2 MgCl_2_, 10 glucose, and 10 HEPES, pH 7.4], test compounds were added. The osmolarity was adjusted to 314 mOsm with sucrose. The recording pipettes contained (in mM) 140 KCl, 10 NaCl, 1 MgCl_2_, 0.5 CaCl_2_, 2 Na_2_-ATP, 5 EGTA, 10 HEPES, and 10 sucrose, pH 7.2. Osmolarity was adjusted to 310 mOsm with sucrose. Only neurons with a membrane potential more negative than −45 mV were included. At resting potential, APs were elicited by current injection through the recording pipette (in 50 pA steps, pulse duration 5 ms, interpulse interval 2 s) until a typical AP was elicited. This protocol was repeated every 2 min before and within 3–15 min after the bath application of the compounds. In addition, a ramp current (0 pA to 3× threshold current) was applied for 500 ms, and the latency of the first AP and the number of evoked APs were measured.

After control recordings, test compounds were added to the bath: baricitinib at a concentration of 0.1 or 1 µM, IL-6 (1 ng/mL) plus its soluble receptor (sIL-6R, 1 ng/mL), Sta21 (10 µM). See Results for detailed protocols.

### 4.6. Statistics

The effects of compounds on nerve fiber recordings in vivo were tested using the Wilcoxon matched-pairs signed-rank test (a non-parametric test that does not require equal distribution), which allows analysis of whether neuronal responses change after application of a compound compared to baseline. Statistical differences in patch-clamp recordings were analyzed using paired *t*-test and *t*-test. Significance was accepted at *p* < 0.05.

## Data Availability

The original contributions presented in the study are included in the article/Supplementary Material. Further inquiries can be directed to the corresponding author.

## Supplementary Materials

The following supporting information can be downloaded at https://www.mdpi.com/article/10.3390/ijms252211943/s1.
