# Supplementary material for: Direct Effects of the Janus Kinase Inhibitor Baricitinib on Sensory Neurons
Source: Int J Mol Sci. 2024 Nov 6;25(22):11943. doi: 10.3390/ijms252211943 (PMC11593535; doi:10.3390/ijms252211943)
Supplement: Supplementary file 1 [file ijms-25-11943-s001.zip › ijms-3287469-supplementary.pdf]

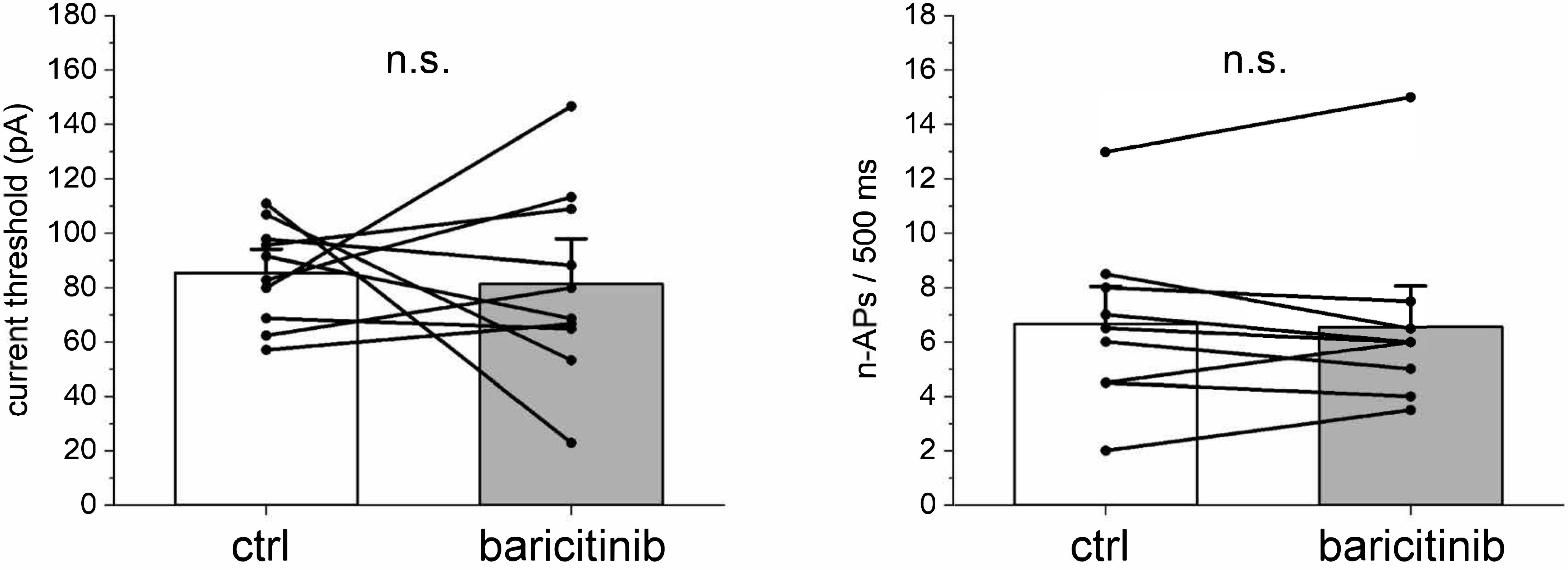

Supplementary Figure S1. Baricitinib alone does not affect current threshold or number of APs in isolated mouse DRGs. Left diagram: Comparison of current thresholds to evoke APs under control conditions and after application of baricitinib (1 μM) for 6 min. Right diagram: Comparison of the number of APs (n-APs) under control conditions and after application of baricitinib (1 μM) for 6 min. No statistical significance shown, paired t-test, n = 10. n.s., not significant.
